# Supplementary material for: Food Insecurity Is Associated with the Quality of Diet of Non-Institutionalized Older Adults from a Southern Chilean Commune: A Cross-Sectional Study
Source: Nutrients. 2021 Dec 23;14(1):36. doi: 10.3390/nu14010036 (PMC8746456; doi:10.3390/nu14010036)
Supplement: Supplementary file 1 [file nutrients-14-00036-s001.zip › nutrients-1509878-supplementary tables.pdf]

Supplementary Table S1. Equivalences of portions (grams) and homemade measures

Food group	Grams per portion	Portion in homemade measure
**Cereal and derivates**		
Cooked rice	130 g	¾ cup
Cooked pasta	130 g	¾ cup
Marraqueta bread	50 g	½ unit
White bread (manufactured)	60 g	2 ½ slices
Sweetened breakfast cereal	40 g	1 cup
High-fiber unsweetened breakfast cereal	50 g	1 ¼ cup
Puffed rice	70 g	2 cups
Crackers	40 g	7 units
**Vegetables**		
Lettuce	50 g	1 cup
Cucumber salad	100 g	½ unit
Cabbage	50 g	1 cup
Arugula	50 g	1 cup
Raw spinach	50 g	1 cup
Tomato	120 g	1 regular unit
Grated raw carrot	60 g	1 cup
Raw onions	60 g	¾ cup
Green beans	70 g	¾ cup
**Fruits and juice**		
Apple	100 g	1 small unit
Pear	100 g	1 small unit
Orange	120 g	1 regular unit
Banana	60 g	½ unit
Peach	130 g	1 regular unit
**Dairy and derivates**		
Milk whole/skimmed/semi-skimmed	200 ml	1 cup
Milk powder whole/skimmed/semi-skimmed	20 g	1 tablespoon
Yoghurt	125 g	1 unit (regular pot)
Quesillo and fresh cheese	90 g	1 slice (4 cm)
Chanco, *mantecoso*, and gouda cheese	30 g	2 thin slices

Cream cheese	30 g	1 ½ tablespoon
**Fats and oils**		
Maravilla, corn, soy, olive, and grape seed oil	5 g	1 teaspoon
Margarine	6 g	1 teaspoon
Mayonnaise	12 g	1 tablespoon
Light mayonnaise	18 g	1 ½ tablespoon
**Meats and fish**		
Low-fat		
Bovine (*Abastero*, stake, loin, black post, entrails, tartar, among others)	50 g	1 piece (6 x 6 x 1 cm)
Chicken	50 g	1 piece (6 x 6 x 1 cm)
Turkey breast (cooked or uncooked)	50 g<br>60 g	1 piece (6 x 6 x 1 cm)<br>Or 3 slices
Pork loin, pork pulp	50 g	1 piece (6 x 6 x 1 cm)
Tuna and mackerel	60 g	1/3 cup
Fish (*Reineta*, conger, corvina, jurel, *lenguado*, *merluza*, *pejegallo*, *pejerrey*, tila)	80 g	1 piece (10 x 6 x 1 cm)
Seafood (shrimp, ribbed mussels, mussels)	80 g	8 units
Eggs	75 g	1,5 units
High-fat		
Bovine (ossobuco, *huachalomo*, vetted loin, strip roast, *plateada*, top sirloin, butcher roast, ribs)	50 g	1 piece (6 x 6 x 1 cm)
Pork chop and pork casserole	50 g	1 piece (6 x 6 x 1 cm)
Fish in oil (Tuna, jurel)<br>Fish (salmon, *cojinova*, trout, jurel, grouper, sawfish)	50 g<br>80 g	1/3 cup<br>1 piece (10 x 6 x 1 cm)
Ribbed mussels in oil<br>Mussels in oil	60 g<br>60 g	6 units<br>9 units
**Legumes and dry fruit**		
Cooked legumes	140 g	¾ cup
Almonds<br>Hazelnuts<br>Peanuts<br>Pistachios<br>Chestnuts	8 g<br>8 g<br>8 g<br>8 g<br>8 g	8 units<br>6 units<br>10 units<br>10 units<br>5 units
**Sausages and cold meats**		
Mortadella	40 g	2 ½ slices

Ham	40 g	1 ¼ slice
Spanish sausage	30 g	¼ unit
Chicken nuggets	50 g	2 ½ units
Pate	30 g	2 heaped tablespoons
Hotdog (chicken, turkey, pork)	50 g	1 unit
**Sweets**		
Ice cream	100 ml	½ cup
Honeybee	5 g	1 teaspoon
Cacao powder	5 g	1 teaspoon
Cakes, Berliner, sponge cake	40 g	1 unit
Chocolates	30 g	2 squares
**Others**		
Sugary drink	200 ml	1 glass
Fried processed foods (*sopaipillas*, chips)	80 g	1 commercial bag
Home-made French chips	150 g	1 cup
Salted snacks (Industrialized snacks, *ramitas*)	25 g	2 cups

Reference: Ratner, R.; Aicardi, V.; Allende, J. *Manual de porciones de intercambio para Chile*; 2nd ed.; Santiago de Chile, 2021.

Supplementary Table S2. Score by food group of the Healthy Eating Index

Food group	Maximum score of 10	Score of 6	Score of 2.5	Score of 0
**Daily consumption**				
1. Cereals and derivatives	Daily	Weekly	Occasional	Never or almost never
2. Vegetables	Daily	Weekly	Occasional	Never or almost never
3. Fruits and juice	Daily	Weekly	Occasional	Never or almost never
4. Dairy and derivates	Daily	Weekly	Occasional	Never or almost never
5. Fats and oils	Daily	Weekly	Occasional	Never or almost never
**Weekly consumption**				
6. Meats and fish	Weekly	Occasional	Daily	Never or almost never
7. Legumes and dry fruit	Weekly	Occasional	Daily	Never or almost never
**Occasional consumption**				
8. Sausages and cold meats	Never or almost never	Occasional	Weeklly	Daily
9. Sweets	Never or almost never	Occasional	Weekly	Daily
10. Others	Never or almost never	Occasional	Weekly	Daily
