# Supplementary material for: Food Insecurity Is Associated with the Quality of Diet of Non-Institutionalized Older Adults from a Southern Chilean Commune: A Cross-Sectional Study
Source: Nutrients. 2021 Dec 23;14(1):36. doi: 10.3390/nu14010036 (PMC8746456; doi:10.3390/nu14010036)
Supplement: Supplementary file 1 [file nutrients-14-00036-s001.zip › Supplementary material - survey.pdf]

SURVEY ON DEMOGRAPHIC AND CLINICAL CHARACTERISTICS, DIET QUALITY AND FOOD INSECURITY IN NON-INSTITUTIONALIZED OLDER ADULTS

Dear older adult, the survey to be carried out will be in strict confidentiality regulations, please be able to respond conscientiously.

SOCIODEMOGRAPHIC BACKGROUND

Name and surname____

National identification document (with check digit)____

Gender

- ○ Female
- ○ Male

¿ Who do you live with?

- ○ Alone
- ○ Nuclear biparental family
- ○ Nuclear monoparental family
- ○ Extended biparental family
- ○ Extended monoparental family

¿ What is the educational level of the main breadwinner in the household?

- ○ No formal studies
- ○ Basic incomplete
- ○ Complete basic
- ○ Humanistic scientific media or incomplete professional technical media, incomplete humanities
- ○ Humanistic Scientific Half or Full Professional Technical Half, Full Humanities
- ○ Technical institute (CFT) or incomplete professional institute (careers 1-3 years)
- ○ Technical institute (CFT) or incomplete professional institute (careers 1 to 3 years); even officer of the Armed Forces and Carabinieri
- ○ Incomplete university (degrees of 4 or more years)
- ○ Complete university; Air Force Officer and Carabinieri

○ Postgraduate (postgraduate, master, doctorate)

Which of the following occupations corresponds to that of the main breadwinner in the household?

○ Unskilled jobs in sales and services, laborers, agriculture, forestry, construction, etc.

○ Workers, operators and artisans of mechanical arts and other trades

○ Trade and market service workers and vendors

○ Farmers and skilled agricultural and fisheries workers

○ Plant and machine operators and vehicle fitters / drivers

○ Public and private office employees

○ Technicians and mid-level professionals (includes NCOs of the Armed Forces and Police)

○ Professionals, scientists and intellectuals

○ Other unidentified groups (includes rentiers, disabled, pensioners, etc.)

NOW TALKING ABOUT YOUR HOME:

Including yourself, how many people live in your household today? Don't consider domestic service, even indoors.____

____

____

Please think about the total income of your household in an average month, considering the contribution of all its members and other additional income such as property income, pensions or pensions. In which of these ranges is the total monthly income of your household? [Apply section according to answer in P3.]

○ 1

○ 2

○ 3

○ 4

○ 5

○ 6

○ 7

Date of birth (Day/Month/Year) ____

ANTHROPOMETRY

Weight (Kg)

Height (M)

Waist circumference (cm)

FOOD INSECURITY SCALE

Have you or someone else in your household worried about not having enough food to eat due to lack of money or other resources?

- ◯ No
- ◯ Yes
- ◯ Does not know
- ◯ Does not respond

Still thinking back to the past 12 months, was there ever a time when you or someone else in your household was unable to eat healthy and nutritious food due to lack of money or other resources?

- ◯ No
- ◯ Yes
- ◯ Does not know
- ◯ Does not respond

Has there ever been a time when you or someone else in your household ate a small variety of foods due to lack of money or other resources?

- ◯ No
- ◯ Yes
- ◯ Does not know
- ◯ Does not respond

Has there ever been a time when you or someone else in your household had to skip breakfast, lunch, or dinner because there weren't enough money or other resources to get food?

- ◯ No
- ◯ Yes
- ◯ Does not know

- Does not respond

Still thinking about the last 12 months, was there ever a time when you or someone else in your household ate less than you thought you should because of lack of money or other resources?

- No
- Yes
- Does not know
- Does not respond

Has your household ever run out of food due to lack of money or other resources?

- No
- Yes
- Does not know
- Does not respond

Has there ever been a time when you or someone else in your household felt hungry but did not eat because there was not enough money or other resources to get food?

- No
- Yes
- Does not know
- Does not respond

Has there ever been a time when you or someone else in your household stopped eating for a whole day due to lack of money or other resources?

- No
- Yes
- Does not know
- Does not respond

FOOD CONSUMPTION FREQUENCY QUESTIONNAIRE

Intake during the last 7 days

Do you consume cereals and derivatives (rice, pasta, bread, breakfast cereals among others)?

- ◯ Daily consumption
- ◯ Weekly
- ◯ Occasional
- ◯ Never or almost never

How many servings of cereals and derivatives (rice, pasta, bread, breakfast cereals among others) do you consume?

- ◯ 1-3 servings
- ◯ 4-5 servings
- ◯ > 6 servings

Do you eat vegetables (cooked and raw)?

- ◯ Daily consumption
- ◯ Weekly
- ◯ Occasional
- ◯ Never or almost never

How many servings of greens and vegetables (cooked and raw) do you eat?

- ◯ 1-3 servings
- ◯ 4-5 servings
- ◯ > 6 servings

Do you eat Fruits (whole and juice)?

- ◯ Daily consumption
- ◯ Weekly
- ◯ Occasional
- ◯ Never or almost never

How many servings of fruits do you consume (whole and juice)?

- 1-3 servings
- 4-5 servings
- > 6 servings

Do you consume milk and derivatives (whole, low-fat, skim milk, yogurt, fresh and fatty cheese)?

- Daily consumption
- Weekly
- Occasional
- Never or almost never

How many servings of milk and dairy products (whole, low-fat, skim milk, yogurt, fresh and fatty cheese) do you consume?

- 1-3 servings
- 4-5 servings
- > 5 servings

Do you eat fat (olive oil, sunflower, butter, margarine)?

- Daily consumption
- Weekly
- Occasional
- Never or almost never

How many servings of fat (olive oil, sunflower, butter, margarine) do you eat?

- 1-3 servings
- 4-5 servings
- > 6 servings

Do you eat meat and fish (eggs, lean and fatty meat, fresh or canned fish)?

- Daily consumption
- Weekly

- Occasional
- Never or almost never

How many servings of meat and fish (eggs, lean and fatty meat, fresh or canned fish) do you eat?

- 1-3 servings
- 4-5 servings
- > 6 servings

Do you eat legumes and nuts?

- Daily consumption
- Weekly
- Occasional
- Never or almost never

How many servings of legumes and nuts do you consume?

- 1-3 servings
- 4-5 servings
- > 6 servings

Do you eat cold cuts and sausages?

- Daily consumption
- Weekly
- Occasional
- Never or almost never

How many portions of cold cuts and sausages do you consume?

- 1-3 servings
- 4-5 servings
- > 6 servings

Do you consume sweet or pastry products (desserts, ice creams, chocolate, delicacies, pastries)?

- ◯ Daily consumption
- ◯ Weekly
- ◯ Occasional
- ◯ Never or almost never

How many servings of sweets or pastries do you eat?

- ◯ 1-3 servings
- ◯ 4-5 servings
- ◯ > 6 servings

Do you consume sugary drinks (flavored boxed milk, regular soda, regular nectar, powdered juices, tea, coffee, mate with sugar)?

- ◯ Daily consumption
- ◯ Weekly
- ◯ Occasional
- ◯ Never or almost never

How many servings of sugary drinks do you consume? (1 glass or 1 box of 200cc equals 1 serving)

- ◯ 1-2 servings
- ◯ 3-4 servings
- ◯ > 5 servings
- ◯ Other

Do you eat fast food (sopaipillas, french fries, hot dogs, hamburgers, churrasco, etc.)?

- ◯ Daily consumption
- ◯ Weekly
- ◯ Occasional
- ◯ Never or almost never

How many servings of fast food do you eat?

○ 1-2 servings

○ 3-4 servings

○ > 5 servings

FOOD ACCESS, CONSUMPTION AND PREFERENCES

Who does the grocery shopping in your home?

○ You

○ Husband / Partner

○ Sons Daughters

○ Other family

When you buy food at home, where do you buy it?

○ Neighborhood warehouse

○ Warehouse style supermarket

○ Normal supermarket

○ Open air fair

○ Purchases to individuals¿

Are you a beneficiary of the food products delivered by PACAM (milk drink, golden year cream)?

○ No

○ Yes

○ Does not know

○ Does not respond

If you are a beneficiary, in what preparations do you use PACAM products?

○ Creams and soups

○ Stews and dishes

○ Cakes and pastries

○ Naturally (milk)

- ⬭ Does not consume
- ⬭ Another way of consumption

VARIOUS

Do you take medication?

- ⬭ No
- ⬭ Yes
- ⬭ Does not know
- ⬭ Does not respond

How many medications do you take in the day?

- ⬭ 1-2
- ⬭ 3-5
- ⬭ 6-8
- ⬭ >8

About how many hours of sleep do you have?

- ⬭ 8-10 hours
- ⬭ 5-7 hours
- ⬭ < 5 hours

Do you practice any kind of physical activity?

- ⬭ No
- ⬭ Yes
- ⬭ Sometimes

The surveyor thanks the participant for their kind participation.
